# Supplementary material for: Disability, Job Satisfaction, and Workplace Accommodations: Evidence from the Healthcare Industry
Source: J Occup Rehabil. 2025 Jul 28;36(3):824–37. doi: 10.1007/s10926-025-10316-0 (PMC13364834; doi:10.1007/s10926-025-10316-0)
Supplement: Supplementary file 1 — Supplementary file1 (DOCX 58 KB) [file 10926_2025_10316_MOESM1_ESM.docx]

Appendix Table 1. Sample Statistics on Disability Status and Disclosure Rates by Disability Type

|  | *Number*  *of respondents*  *with disabilities* | *Percent*  *of respondents*  *with disabilities* |
| --- | --- | --- |
| *Types of Disabilities (Not Mutually Exclusive)* | 228 | 100.0 |
| Deaf/difficulty hearing | 33 | 14.5 |
| Blind/difficulty seeing | 10 | 4.4 |
| Difficulty concentrating/making decisions | 113 | 49.6 |
| Difficulty walking/climbing stairs | 57 | 25.0 |
| Difficulty dressing/bathing | 7 | 3.1 |
| Difficulty doing errands alone | 39 | 17.2 |
| Difficulty interacting with others | 47 | 20.6 |
| Long-term health impairment | 106 | 46.5 |
|  |  |  |
| *Disclosure Among People with Disabilities* | 228 | 100.0 |
| Have you disclosed your health condition, impairment, or  disability to your employer? | | |
| Yes | 117 | 51.8 |
| No | 57 | 25.2 |
| It's complicated | 52 | 23.0 |
| Did not respond | 2 | 0.9 |
|  |  |  |
| *Disclosure Rates by Disability Type* |  |  |
| Yes | 117 | 51.8 |
| Deaf/difficulty hearing | 19 | 57.6 |
| Blind/difficulty seeing | 4 | 40.0 |
| Difficulty concentrating/making decisions | 46 | 40.7 |
| Difficulty walking/climbing stairs | 39 | 68.4 |
| Difficulty dressing/bathing | 3 | 42.9 |
| Difficulty doing errands alone | 19 | 48.7 |
| Difficulty interacting with others | 19 | 40.4 |
| Long-term health impairment | 65 | 61.3 |
| No | 57 | 25.2 |
| Deaf/difficulty hearing | 10 | 30.3 |
| Blind/difficulty seeing | 4 | 40.0 |
| Difficulty concentrating/making decisions | 32 | 28.3 |
| Difficulty walking/climbing stairs | 8 | 14.0 |
| Difficulty dressing/bathing | 4 | 57.1 |
| Difficulty doing errands alone | 11 | 28.2 |
| Difficulty interacting with others | 15 | 31.9 |
| Long-term health impairment | 17 | 16.0 |
| It’s complicated | 52 | 23.0 |
| Deaf/difficulty hearing | 4 | 12.1 |
| Blind/difficulty seeing | 2 | 20.0 |
| Difficulty concentrating/making decisions | 34 | 30.1 |
| Difficulty walking/climbing stairs | 9 | 15.8 |
| Difficulty dressing/bathing | 0 | 0.0 |
| Difficulty doing errands alone | 8 | 20.5 |
| Difficulty interacting with others | 13 | 27.7 |
| Long-term health impairment | 23 | 21.7 |
| Did not respond | 2 | 0.9 |

Note: Sample size: 228 persons with a disability. Types of disability are not mutually exclusive.

Appendix Table 2. Sample Means for Demographic Characteristics by Disclosed Disability Status (Measured as Proportions Unless Indicated Otherwise)

|  | *Disclosed*  *Disability* | *Undisclosed/*  *No Disability* |  |
| --- | --- | --- | --- |
| *Demographic Characteristic* | *Mean* | *Mean* | *Difference* |
| Age | 45.468 | 44.530 | 0.938 |
| # Children at home | 1.809 | 2.010 | -0.202^*^ |
| Gender |  |  |  |
| Man | 0.079 | 0.106 | -0.027 |
| Woman | 0.877 | 0.861 | 0.016 |
| Nonbinary | 0.044 | 0.032 | 0.011 |
| Race/Ethnicity |  |  |  |
| Black | 0.112 | 0.120 | -0.008 |
| White | 0.871 | 0.832 | 0.038 |
| Hispanic | 0.052 | 0.027 | 0.025 |
| American Indian/Alaska Native | 0.026 | 0.042 | -0.016 |
| Asian/Pacific Islander | 0.009 | 0.017 | -0.009 |
| Multiracial/other | 0.060 | 0.060 | 0.000 |
| Married | 0.482 | 0.643 | -0.161^***^ |
| Education |  |  |  |
| <9th grade | 0.000 | 0.001 | -0.001 |
| High school graduate | 0.121 | 0.069 | 0.051^**^ |
| Some college, no degree | 0.207 | 0.155 | 0.052 |
| Associate degree | 0.155 | 0.178 | -0.023 |
| Bachelor’s degree | 0.336 | 0.360 | -0.024 |
| Master’s degree | 0.147 | 0.178 | -0.032 |
| Professional degree/PhD | 0.034 | 0.058 | -0.023 |
| Income >=$75,000 | 0.216 | 0.348 | -0.132^***^ |
| Works full-time | 0.872 | 0.885 | -0.013 |
| Worked at employer before pandemic | 0.726 | 0.794 | -0.068^*^ |
| Worked at employer <= 5 years | 0.547 | 0.410 | 0.137^***^ |
| Occupation |  |  |  |
| Administrative support staff | 0.216 | 0.176 | 0.040 |
| Professional/technical staff | 0.138 | 0.131 | 0.007 |
| Nurse | 0.198 | 0.255 | -0.056 |
| Physician | 0.000 | 0.007 | -0.007 |
| Healthcare aide | 0.078 | 0.046 | 0.032 |
| Other healthcare provider | 0.233 | 0.210 | 0.023 |
| Manager | 0.086 | 0.121 | -0.035 |
| Other service provider | 0.052 | 0.055 | -0.003 |

Note: This Appendix Table reports sample means for the disability gap when the disability sample is narrowed to those who disclosed their disability to their employer. Sample size 993. *** statistically significant at 1%, ** at 5%, and * at 10% in 2-tail t tests. Results denote the proportion of respondents who gave the indicated response in the survey, ranging from 0 to 1.

Appendix Table 3. Sample Means for Accommodation Requests by Disclosed Disability Status (Measured as Proportions)

|  | *Disclosed*  *Disability* | *Undisclosed/*  *No Disability* |  |
| --- | --- | --- | --- |
| *Accommodations* | *Mean* | *Mean* | *Difference* |
| Have you ever requested accommodations? | 0.730 | 0.574 | 0.156^***^ |
| Type requested: equipment | 0.452 | 0.476 | -0.024 |
| Type requested: physical change to workplace | 0.333 | 0.256 | 0.077 |
| Type requested: work from home | 0.298 | 0.278 | 0.020 |
| Type requested: change to work schedule | 0.679 | 0.566 | 0.113^*^ |
| Type requested: restructure job | 0.262 | 0.198 | 0.064 |
| Type requested: move to another job or location | 0.262 | 0.142 | 0.120^***^ |
| Type requested: change communications/info sharing | 0.560 | 0.454 | 0.106^*^ |
| Type requested: other | 0.274 | 0.184 | 0.090^*^ |
| Most recent request: equipment | 0.151 | 0.211 | -0.060 |
| Most recent request: physical change to workplace | 0.058 | 0.058 | 0.000 |
| Most recent request: work from home | 0.070 | 0.066 | 0.004 |
| Most recent request: change to work schedule | 0.302 | 0.281 | 0.021 |
| Most recent request: restructure job | 0.116 | 0.076 | 0.041 |
| Most recent request: move to another job or location | 0.081 | 0.058 | 0.024 |
| Most recent request: change communications/info sharing | 0.140 | 0.179 | -0.040 |
| Most recent request: other | 0.081 | 0.072 | 0.010 |
| Most recent accommodation was requested within the past 12 months | 0.786 | 0.826 | -0.040 |
| Did you request this change in order to accommodate any health condition, impairment, or disability that you may have? | 0.393 | 0.104 | 0.289^***^ |
| Was the requested change or accommodation made? |  |  |  |
| Yes | 0.512 | 0.477 | 0.035 |
| No | 0.262 | 0.297 | -0.035 |
| Partially | 0.226 | 0.226 | 0.000 |

Note: This Appendix Table reports sample means for the disability gap when the disability sample is narrowed to those who disclosed their disability to their employer. Results denote the proportion of respondents who gave the indicated response in the survey, ranging from 0 to 1. Sample size 993 for first question. Responses for remaining questions are conditional on having ever requested accommodations. *** statistically significant at 1%, ** at 5%, and * at 10% in 2-tail t tests.

Appendix Table 4. Detailed Indicators and Sample Means for Job Experiences by Disability Status (Measured as Proportions)

|  | *Self-Reported* | | | *Disclosed* | | |
| --- | --- | --- | --- | --- | --- | --- |
|  | *Disb.* | *No Disb.* | *Disb.* | *Disb.* | *NoDisb/Undisc* | *Disb.* |
| *% of People Who Agree with These Statements:* | *Mean* | *Mean* | *Diff.* | *Mean* | *Mean* | *Diff.* |
| **Somewhat or very satisfied in job** | **0.592** | **0.648** | **-0.056** | **0.590** | **0.641** | **-0.051** |
| **Index of agreement on job autonomy** | **0.575** | **0.599** | -**0.023** | **0.593** | **0.593** | **0.001** |
| Currently pandemic job allows me to decide when to begin and end work each day | 0.356 | 0.391 | -0.035 | 0.365 | 0.385 | -0.020 |
| Currently I have some control over the sequencing of my work activities | 0.704 | 0.744 | -0.040 | 0.707 | 0.738 | -0.031 |
| Currently I can decide when to do particular work activities | 0.665 | 0.665 | 0.000 | 0.707 | 0.659 | 0.048 |
| **Index of turnover intentions** | **0.420** | **0.323** | **0.096**^***^ | **0.407** | **0.337** | **0.070**^*^ |
| Currently I plan to look for job outside this company during the next year | 0.363 | 0.283 | 0.080^**^ | 0.359 | 0.294 | 0.065 |
| Currently I often think about quitting my job at this company | 0.439 | 0.334 | 0.104^***^ | 0.419 | 0.350 | 0.069 |
| Currently I want to get a new job | 0.461 | 0.353 | 0.108^***^ | 0.444 | 0.368 | 0.076 |
| **Index of organizational commitment** | **0.459** | **0.530** | -**0.071^**^** | **0.481** | **0.518** | -**0.037** |
| I feel a strong sense of 'belonging' to the employer | 0.469 | 0.577 | -0.108^***^ | 0.470 | 0.563 | -0.093^*^ |
| I feel like 'part of the family' at the employer | 0.427 | 0.526 | -0.099^***^ | 0.436 | 0.513 | -0.077 |
| The employer has a great deal of personal meaning for me | 0.476 | 0.487 | -0.011 | 0.538 | 0.477 | 0.061 |
| **Index of organizational citizenship behaviors** | **0.550** | **0.557** | -**0.007** | **0.584** | **0.551** | **0.033** |
| I keep up with developments at the employer | 0.610 | 0.634 | -0.024 | 0.615 | 0.630 | -0.014 |
| I offer ideas to improve the functioning of the employer | 0.430 | 0.450 | -0.020 | 0.453 | 0.444 | 0.009 |
| I take action to protect the employer from potential problems | 0.610 | 0.588 | 0.022 | 0.684 | 0.581 | 0.103^**^ |
| **Index of perceived organizational support** | **0.309** | **0.403** | -**0.094**^***^ | **0.376** | **0.383** | -**0.006** |
| The employer really cares about my well-being | 0.339 | 0.433 | -0.093^**^ | 0.371 | 0.417 | -0.046 |
| The employer takes pride in my accomplishments at work | 0.313 | 0.412 | -0.100^***^ | 0.405 | 0.387 | 0.018 |
| The employer cares about my opinions | 0.276 | 0.366 | -0.090^**^ | 0.353 | 0.344 | 0.009 |

Continued on next page

Appendix Table 4 Continued. Detailed Indicators and Sample Means for Job Experiences, by Disability Status

|  | *Self-Reported* | | | *Disclosed* | | |
| --- | --- | --- | --- | --- | --- | --- |
|  | *Disb.* | *No Disb.* | *Disb.* | *Disb.* | *NoDisb/Undisc* | *Disb.* |
| *% of People Who Agree with These Statements:* | *Mean* | *Mean* | *Diff.* | *Mean* | *Mean* | *Diff.* |
| **Index of employer openness to differences** | **0.453** | **0.561** | -**0.107**^***^ | **0.490** | **0.542** | -**0.052** |
| The employer has a non-threatening environment in which people can reveal their 'true' selves | 0.482 | 0.616 | -0.133^***^ | 0.521 | 0.594 | -0.072 |
| Employees are valued for who they are as people, not just for the jobs that they fill | 0.355 | 0.450 | -0.094^**^ | 0.402 | 0.432 | -0.030 |
| We have a culture in which employees appreciate the differences that people bring to the workplace | 0.522 | 0.616 | -0.095^**^ | 0.547 | 0.601 | -0.054 |
| **Index of climate for inclusion** | **0.348** | **0.416** | -**0.069**^**^ | **0.385** | **0.403** | -**0.018** |
| Employee input is actively sought | 0.390 | 0.463 | -0.072^*^ | 0.402 | 0.452 | -0.050 |
| Everyone's opinions for how to do things better are given serious consideration | 0.298 | 0.343 | -0.045 | 0.333 | 0.333 | 0.001 |
| Employees' insights are used to rethink or redefine work practices | 0.325 | 0.399 | -0.075^**^ | 0.359 | 0.385 | -0.026 |
| Management exercises the belief that problem-solving is improved when input from different roles, ranks, and functions is considered | 0.377 | 0.463 | -0.086^**^ | 0.444 | 0.443 | 0.001 |
| **Index of treatment of people with disabilities** | **0.415** | **0.438** | -**0.023** | **0.459** | **0.429** | **0.030** |
| Employees with disabilities have the same opportunities as people without disabilities | 0.469 | 0.496 | -0.027 | 0.521 | 0.486 | 0.036 |
| The employer is making strong efforts to improve conditions and opportunities for people with disabilities | 0.382 | 0.439 | -0.058 | 0.419 | 0.427 | -0.008 |
| The environment is such that, when accommodations are made, people with disabilities can be just as productive as people without disabilities | 0.447 | 0.489 | -0.041 | 0.513 | 0.475 | 0.038 |
| Bias against people with disabilities exists where I work | 0.259 | 0.122 | 0.136^***^ | 0.291 | 0.135 | 0.155^***^ |
| Where I work, employees without disabilities are treated better than employees with disabilities | 0.137 | 0.067 | 0.070^***^ | 0.162 | 0.072 | 0.090^***^ |
| Top management commits to hire people with disabilities | 0.138 | 0.174 | -0.036 | 0.148 | 0.168 | -0.021 |
| Employees treat people with disabilities with respect | 0.586 | 0.679 | -0.093^***^ | 0.624 | 0.662 | -0.039 |
| My manager treats people with disabilities with respect | 0.659 | 0.702 | -0.042 | 0.707 | 0.690 | 0.017 |
| The employer is responsive to the needs of people with disabilities | 0.471 | 0.532 | -0.061 | 0.513 | 0.519 | -0.006 |
| My manager is responsive to the needs of people with disabilities | 0.608 | 0.672 | -0.064^*^ | 0.692 | 0.652 | 0.040 |

Appendix Table 4 Continued. Detailed Indicators and Sample Means for Job Experiences, by Disability Status

|  | *Self-Reported* | | | *Disclosed* | | |
| --- | --- | --- | --- | --- | --- | --- |
|  | *Disb.* | *No Disb.* | *Disb.* | *Disb.* | *NoDisb/Undisc* | *Disb.* |
| *% of People Who Agree with These Statements:* | *Mean* | *Mean* | *Diff.* | *Mean* | *Mean* | *Diff.* |
| **Index of manager relations (leader-member exchange)** | **0.657** | **0.761** | -**0.105**^***^ | **0.706** | **0.742** | -**0.036** |
| I usually know how satisfied my manager is with what I do | 0.699 | 0.801 | -0.101^***^ | 0.726 | 0.784 | -0.058 |
| I feel that my manager understands my problems and needs | 0.562 | 0.712 | -0.150^***^ | 0.615 | 0.686 | -0.070 |
| I feel that my manager recognizes my potential | 0.611 | 0.750 | -0.140^***^ | 0.650 | 0.728 | -0.078^*^ |
| I can count on my manager to support me even when I'm in a tough situation at work | 0.668 | 0.755 | -0.086^***^ | 0.718 | 0.737 | -0.019 |
| I would support my manager's decision even if he or she was not present | 0.752 | 0.801 | -0.049 | 0.821 | 0.786 | 0.035 |
| I have an effective working relationship with my manager | 0.730 | 0.821 | -0.091^***^ | 0.769 | 0.804 | -0.035 |
| If necessary, my manager would use his or her power and influence to help me | 0.575 | 0.692 | -0.117^***^ | 0.641 | 0.669 | -0.028 |
| **Index of coworker relations (coworker exchange)** | **0.706** | **0.801** | -**0.095**^***^ | **0.698** | **0.790** | -**0.092**^***^ |
| I usually know how satisfied my coworkers are with what I do | 0.681 | 0.804 | -0.123^***^ | 0.672 | 0.790 | -0.117^***^ |
| I feel that my coworkers understand my problems and needs | 0.590 | 0.711 | -0.121^***^ | 0.595 | 0.695 | -0.100^**^ |
| I can count on my coworkers to support me even when I'm in a tough situation at work | 0.740 | 0.818 | -0.078^***^ | 0.724 | 0.811 | -0.086^**^ |
| I would support my coworkers' decisions even if they were not present | 0.811 | 0.837 | -0.027 | 0.810 | 0.834 | -0.024 |
| I have an effective working relationship with my coworkers | 0.828 | 0.893 | -0.064^***^ | 0.802 | 0.888 | -0.086^***^ |
| If necessary, my coworkers would use their power and influence to help me | 0.586 | 0.744 | -0.158^***^ | 0.586 | 0.724 | -0.138^***^ |

Note: This Appendix Table reports sample means for the disability gap when using the sample of people who reported a disability in the survey (columns 1-3), and the smaller sample who disclosed their disability to their employer (columns 4-6). Sample size 993. *** statistically significant at 1%, ** at 5%, and * at 10% in 2-tail t tests. Results denote the proportion of respondents who agree with the statements, ranging from 0 to 1. Indicators in each index are weighted equally.

Appendix Table 5. Regression-Adjusted Disability Gaps in Perceptions of Work Experiences

|  | *Regression-Adjusted* |
| --- | --- |
|  | *Disability Gap* |
| Somewhat or very satisfied in job | -0.047 |
|  | (0.038) |
| Index of agreement on job autonomy | 0.015 |
|  | (0.028) |
| Index of turnover intentions | 0.095^***^ |
|  | (0.033) |
| Index of employee organizational commitment | -0.056^*^ |
|  | (0.033) |
| Index of employee organizational citizenship behaviors | 0.015 |
|  | (0.030) |
| Index of perceived organizational support | -0.085^**^ |
|  | (0.033) |
| Index of employer openness to differences | -0.098^***^ |
|  | (0.033) |
| Index of climate for inclusion | -0.055^*^ |
|  | (0.033) |
| Index of treatment of people with disabilities | -0.013 |
|  | (0.023) |
| Index of relationship with manager (leader-member exchange) | -0.095^***^ |
|  | (0.027) |
| Index of relationships with coworkers (coworker exchange) | -0.067^***^ |
|  | (0.025) |

Note: Sample size 993. ^***^ statistically significant at 1%, ^**^ at 5%, and ^*^ at 10% in 2-tail t tests. Coefficients above are from separate estimations of each work experience measure regressed on a dummy variable for disability plus control variables for age, gender, race/ethnicity, marital status, education, income above $75,000, number of children at home, managerial role, full-time worker, and tenure at the employer.

**Appendix Notes:**

**Sample and Perceptions of Work Experience Scales**

**Sample:**

Our survey instrument included questions on employees’ awareness and perceptions of employer policies that address the physical and mental health needs of workers. It focused on employer practices around work from home, and it also asked about work experiences before the pandemic. Survey questions used the wording “Before March 2020” to denote the period before the pandemic started, and phrases such as “currently” or “today” to denote the current period at the time of the survey. We used the data to calculate simple summary statistics on the prevalence of work from home, disability disclosure, perceptions of workplace inclusiveness, treatment of people with disabilities, and various measures of job satisfaction.

In collaboration with partners from the healthcare system, our research team distributed the survey link via Qualtrics to employees. The distribution included a cover letter providing details about the study and outlining the informed consent process.

Qualtrics reported 1,405 respondents. We dropped 135 of those respondents because they clicked on the survey link but did not answer any questions. An additional 277 respondents did not respond to questions about their disability status, so we also dropped these individuals, leaving a sample of 993. Robustness checks in which these 277 respondents were kept in the sample and assumed to have no disability yielded substantively similar results to those reported in the paper.

**Perceptions of Work Experience Scales:**

Perceived organizational support:

1. The organization really cares about my well-being.
2. The organization takes pride in my accomplishments at work.
3. The organization cares about my opinions.

Cites:

3 items from Wayne et al. based on longer scale from Eisenberger et al.

Wayne, S., Shore, L., & Liden, R. (1997). Perceived organizational support and leader-member exchange: A social exchange perspective. *Academy of management Journal, 40,* 82-111.

Eisenberger, R., Huntington, R., Hutchison, S., & Sowa, D. (1986). Perceived organizational support. *Journal of Applied Psychology, 71,* 500-507.

Organizational commitment:

This is known more specifically as “affective organizational commitment”

1. I feel a strong sense of “belonging” to my organization.
2. I feel like "part of the family" at my organization.
3. My organization has a great deal of personal meaning for me.

Cite:

Drawn from Meyer, J.P, Allen, M.J., & Smith, C.A. (1993). Commitment to organizations and occupations: Extension and test of a three-component conceptualization. *Journal of Applied Psychology, 78,* 538-551.

Organizational citizenship behaviors:

How often do you engage in these behaviors

1. Keep up with developments in the organization.
2. Offer ideas to improve the functioning of the organization.
3. Take action to protect the organization from potential problems.

Cite:

Lee, K. and Allen, N.J. (2002), “Organizational citizenship behavior and workplace deviance: the role of affect and cognitions”, Journal of Applied Psychology, Vol. 87 No. 1, pp. 131-142, doi: 10.1037// 0021-9010.87.1.131.

Leader-member exchange:

Please indicate the extent to which you agree with these statements about the relationship between you and your supervisor/manager? *(Please circle ONE answer for each item)*

I usually know how satisfied my manager is with what I do.

I feel that my manager understands my problems and needs

I feel that my manager recognizes my potential.

I can count on my manager to support me even when I’m in a tough situation at work.

I would support my manager’s decisions even if he or she was not present.

I have an effective working relationship with my manager.

If necessary, my manager would use his or her power and influence to help me.

Adapted from:

Graen, G. B., & Uhl-Bien, M. (1995). Relationship-based approach to leadership: Development of leader-member exchange (LMX) theory of leadership over 25 years: Applying a multi-level multi-domain perspective. *The leadership quarterly*, *6*(2), 219-247.

Coworker exchange:

Please indicate the extent to which you agree with these statements about the relationship between you and your coworkers (Please circle ONE answer for each item) ※

E34. I usually know how satisfied my coworkers are with what I do.

E35. I feel that my coworkers understand my problems and needs.

E36. I can count on my coworkers to support me even when I’m in a tough situation at work.

E37. I would support my coworkers’ decisions even if they were not present.

E38. I have an effective working relationship with my coworkers.

E39. If necessary, my coworkers would use their power and influence to help me.

Cite:

Sherony, K. M., & Green, S. G. (2002). Coworker exchange: relationships between coworkers, leader-member exchange, and work attitudes. *Journal of applied psychology*, *87*(3), 542.

Climate for inclusion:

This is a subscale representing the dimension of climate for inclusion in decision-making

How would you rate the inclusiveness of your organization with regard to employees’ ideas and experiences in general, particularly on each of the following? *Please CHOOSE ONE answer for each item.*

E13. In my organization, employee input is actively sought.

E14. In my organization, everyone’s ideas for how to do things better are given serious consideration.

E15. In my organization, employees’ insights are used to rethink or redefine work practices.

E16. Management exercises the belief that problem-solving is improved when input from different roles, ranks, and functions is considered.

Cite:

Nishii, L. H. (2013). The benefits of climate for inclusion for gender-diverse groups. *Academy of Management journal*, *56*(6), 1754-1774.

Turnover intentions:

C9. I will look for a job outside this company during the next year.

C10. I often think about quitting my job at this company.

C11. I would like to get a new job.

Cite:

Konovsky, M. A., & Cropanzano, R. (1991). Perceived fairness of employee drug testing as a predictor of employee attitudes and job performance. *Journal of applied psychology*, *76*(5), 698.

Autonomy:

C5. The job denies me any chance to use my personal initiative or judgment in carrying out the work.

C6. The job gives me considerable opportunity for independence and freedom in how I do the work.

C7. The job gives me considerable flexibility to work at my personal “peak” times (i.e., the times of day I feel most productive)

C8. I have complete freedom to schedule my own work hours.

These four are all from Desroisers (2001), drawing from Hackman and Oldham (1978) and Breaugh (1985).

Cites:

Breaugh, J. A. (1985). The measurement of work autonomy. *Human relations*, *38*(6), 551-570.

Desrosiers, E. I. (2001). *Telework and work attitudes: The relationship between telecommuting and employee job satisfaction, organizational commitment, perceived organizational support, and perceived co-worker support*. Purdue University.

Hackman, J. R., & Oldham, G. R. (1975). Development of the job diagnostic survey. *Journal of Applied psychology*, *60*(2), 159.

The accommodations questions are all from:

Schur, L., Nishii, L., Adya, M., Kruse, D., Bruyère, S. M., & Blanck, P. (2014). Accommodating employees with and without disabilities. *Human Resource Management*, *53*(4), 593-621.
